# Conceptualization of Empowerment and Pathways Through Which Cash Transfers Work to Empower Young Women to Reduce HIV Risk: A Qualitative Study in Tanzania

**DOI:** 10.1007/s10461-020-02850-0

**Published:** 2020-04-01

**Authors:** Joyce Wamoyi, Peter Balvanz, Kaitlyn Atkins, Margaret Gichane, Esther Majani, Audrey Pettifor, Suzanne Maman

**Affiliations:** 1 National Institute of Medical Research, P.O Box 1462, Mwanza, Tanzania; 2 Department of Health Behavior, Gillings School of Global Public Health, University of North Carolina at Chapel Hill, Chapel Hill, NC 27516 USA; 3 Sauti Program|USAID Grantee, Dar es Salaam, Tanzania; 4 Department of Epidemiology, Gillings School of Global Public Health, University of North Carolina at Chapel Hill, Chapel Hill, NC 27516 USA; 5 Carolina Population Center, University of North Carolina at Chapel Hill, Chapel Hill, NC 27516 USA

**Keywords:** Adolescent girls, Young women, Cash transfers, Empowerment, Tanzania, Sexual and reproductive health

## Abstract

Although cash transfers (CT) are hypothesized to reduce AGYW’s HIV risk, little is known about the mechanisms through which CT empower AGYW. We explored the impact of a CT intervention on AGYW’s sexual decision-making in order to describe the pathways through which the cash may influence risk behavior. The study employed qualitative methods involving: 20 longitudinal in-depth interviews (IDIs), 40 cross-sectional IDIs, 20 narrative IDIs, and two focus group discussions with AGYW ages 15–23 participating in a CT intervention. AGYW’s conceptualized empowerment as: “independence”, “hope and aspiration”. Potential pathways through which CT empowered AGYW were: economic, hope and aspiration for a better future, and access to knowledge. As a result of this empowerment, AGYW reported reductions in transactional sex, experiences of intimate partner violence, and risky-sexual behaviour. A sense of responsibility developed through economic empowerment, enhanced participants’ self-esteem and confidence in decision-making leading to changes in AGYW’s sexual risk behaviors.

## Introduction

Adolescent girls and young women (AGYW) in sub-Saharan Africa make up 10% of the total population, yet they account for 25% of the new HIV infections [1]. Structural drivers such as poverty and gender inequalities increase AGYW’s HIV risk [2]. In order to obtain their basic needs, some AGYW engage in risky sexual behaviours such as multiple and concurrent sexual partnerships, age-disparate sex, transactional sex, and unprotected sex [3–5]. Specifically, AGYW’s engagement in transactional sex has been associated with increased risk of HIV infection [6–8].

Although there has been some progress towards addressing economic vulnerabilities and gender inequalities in Tanzania, many AGYW still live in poverty and have limited decision-making power in their households and sexual relationships [9, 10]. Researchers and advocates have called for empowering AGYW so they are able to manage their lives and improve their access to and control over various material and non-material resources [9, 11]. However, the strategies programmes use to empower women vary from one context to another. Empowerment is both a state and a process that involves enhancing the capacity of individuals to make choices and to transform those choices to desired actions and outcomes. Women’s empowerment entails the improvement in their ability to manage their own lives (e.g. improve their access to education, access to formal sector employment, access to entrepreneurship, access to finance, control over fertility), which may be obtained through increased access to key resources and activities [12].

Cash transfers (CT) are encouraged as an intervention that may empower women to make their own decisions and support themselves financially [13]. They are considered an effective social protection mechanism through provision of cash subsidies to extremely poor households [14] and sometimes directly to individuals [15–17]. While a few studies, mainly in South Africa, have explored how young women use CT [18], little has been done to describe how beneficiaries perceive these cash interventions and whether they consider them as empowering in meaningful ways. We set out to explore the role of a CT intervention in AGYW’s decision-making, and their perceptions of if and how (pathways) the CT empowered them.

## Methods

### Sauti Project

This qualitative study was conducted with AGYW enrolled in the *Sauti* project. The *Sauti* project is one of the implementers of the Determined, Resilient, Empowered, AIDS free, Mentored and Safe (DREAMS) initiative package, a PEPFAR-funded program that combines biomedical, behavioral, and structural interventions to reduce new HIV infections among vulnerable populations. We specifically recruited participants who were recipients of one of the *Sauti* project’s structural interventions, a combined CT and economic empowerment program. This intervention aimed to reduce economic vulnerability among AGYW by enabling them to reduce sexual risk behaviours such as transactional sex, age-disparate sex, concurrent partnerships, and inconsistent condom use.

CT of Tanzanian shillings (TZS) 70,000 (~ USD 31) were provided to program participants every three months over an 18-month period. Cash was delivered via SIM cards on mobile phones, both provided by *Saut*i project. The economic empowerment intervention that accompanied the CT, called WORTH+, included financial literacy education, individual and group savings and loan, and entrepreneurship skills training such as soap making. WORTH+ groups were led by a trusted mentor and met on a weekly basis. In order to receive CT, participants were also required to attend 10 h of behavior change communication (BCC) sessions led by trained peers and designed to address the major determinants of HIV risk, gender-based violence, and reproductive health. The BCC package provided education on HIV and other STI prevention, gender-based violence prevention, family planning, condom use, negotiation skills, self-efficacy/agency skills, and promoted health-seeking behaviors.

To be eligible for the CT, participants had to be: female; between the ages of 15 and 23; resident of the intervention villages; out of school; completed BCC sessions; and, willing and able to give voluntary, informed consent/assent to all study procedures including HIV and HSV-2 testing and receipts of test result. Participants under 18 years of age required the consent of a guardian unless they met criteria for emancipated minors (i.e. minors who are married, pregnant, have given birth, or are heads of household).

### Data Collection

We recruited participants (n = 80) from *Sauti* project sites in the Shinyanga region of Northwest Tanzania, including Bulungwa, Kahama, and Shinyanga municipal. Shinyanga is a primarily agricultural area and is among the poorest regions of Tanzania. We used a multi-methods qualitative approach that included longitudinal in-depth interviews (n = 20), cross sectional in-depth interviews (n = 40), and narrative timeline interviews (n = 20).

We conducted longitudinal interviews (n = 20) to examine patterns of CT use and their impact over a 1-year period. Participants were interviewed in June 2017 just after the first round of CT, then again in June 2018 after they had received three or more payments. All longitudinal interviews were conducted with participants from Shinyanga municipal, since initial CT had only reached this area by June 2017.

We conducted additional cross-sectional interviews (n = 40) in June 2018 in all three recruitment areas with AGYW who had received three or more payments. Interviews broadly assessed how participants spent their CT, to whom they disclosed cash receipt, how those people reacted to the program and any advice they provided, and how this cash may have impacted their relationships. Towards the end of our research, we conducted a community-engaged activity where AGYW (n = 10) who had participated in the CT intervention joined three participatory focus group discussions (FGD) involving reflection on benefits and drawbacks on the CT intervention, with a direct focus on empowerment.

Finally, we conducted narrative timeline interviews with women (n = 20) in all three recruitment areas. Narrative timeline interviews are a participatory research method wherein participants develop visual timelines depicting relationships over a specified amount of time. Timelines have been used as a tool to provide more in-depth discussion of relationship qualities, as well as to allow for comparisons of relationships across time [19, 20]. In our study, timeline interviews were used to understand how AGYW’s sexual behavior and relationships changed over time, and what influence the CT had on that change.

All interviews and FGDs were conducted in Kiswahili by trained female research assistants. Individual interviews and FGDs were conducted in private locations at the civil society organizations implementing the *Sauti* project and near the homes of some participants. All interviews and FGDs were audio recorded, transcribed verbatim in Kiswahili, and translated by trained research assistants. Participants were compensated TZS 5000 (~ USD 2) for transportation costs to the interview. We obtained ethical approval from the institutional review boards (IRBs) of The University of North Carolina and the National Institute for Medical Research in Tanzania. Informed consent was obtained prior to any interview or FGD.

### Analyses

All interviews and FGDs were transcribed verbatim in Kiswahili, and then translated into English. Interview and FGD transcripts were reviewed for quality of transcription and translation. Six researchers coded transcripts using Dedoose, an online qualitative software. We applied thematic analysis to analyze data. For each interview type, one transcript was coded by all analysts, and then coding consistency was compared across analysts. Upon reaching consensus and modifying code definitions, transcripts were coded by multiple analysts. Approximately 20% of the remaining transcripts were quality checked for coding by another analyst. Analysts took notes on any discrepancies in coding found, and then resolved with the group in team meetings. After coding was complete, we used coded transcripts to create matrices summarizing major topical areas, including the impact of CT on relationships and sexual risk. We reviewed matrices to identify patterns within and across interviews.

## Results

The results are structured in three broad themes: emic conceptualization of empowerment, levels of empowerment and their impact on AGYW’s lives, and pathways of empowerment.

### EMIC Conceptualization of Empowerment

AGYW described empowerment in varied but sometimes overlapping ways. These ranged from the act of doing or performing certain activities to a state of being. The majority of AGYW emphasized economic empowerment over sexual empowerment. They linked empowerment to affording material needs, a shift from depending on parents and/or sexual partners for material needs to depending on self, the ability to make decisions in sexual relationships, and having sex on AGYW’s terms. When conceptualizing empowerment as performing certain activities, AGYW used expressions such as: “*I can afford all I want*”, *“we depend on ourselves*”, “*look towards ourselves to sort our financial issues*”, “*make our own decisions*”, and *“move from one point to another”*. Expressions depicting empowerment as a state of being were: *“self-reliant”, “self-aware”* and *“moved out of the box*”.

Despite such variation in their descriptions, AGYW consistently saw “dependency” in contrast to empowerment:

If we say that we’ve been empowered… we’ve moved from dependency to independence… if you depended on a parent for even a small amount of money you would fail to get money for even needs like sanitary pads...but the coming of this organization has really empowered us by giving us this money and us starting these businesses…we now have our own money…We have that ability to be independent, to do what you want without depending on any one. (FGD)

### Empowerment at Different Levels of AGYW’s Lives

AGYW reflected on the impact of empowerment at three levels: community, interpersonal (family and intimate relationships), and intrapersonal. At the community level, empowerment was linked to social standing and was demonstrated by ownership of property, ownership of other material things, and access to money. Not being able to meet one’s needs led to community disrespect; AGYW reflected on past experiences depending on others as de-humanizing. According to the majority of AGYW, the community disrespected people without a job but respected those with a source of income.

The perception of girls has changed. The community disrespects those without jobs, the idlers. The CT has helped me…When I got that money, I entered into business. I am now busy with my business. So, other people [community] see that… I mean if you depend on yourself, the community wouldn’t disrespect you. (IDI, 17-years, single)

At the interpersonal level, CT seemed to have empowered AGYW economically and improved their interactions, both in families and in intimate relationships. Some married participants reported feeling exploited by their spouses prior to the CT, saying that some male partners previously refused to provide for the needs of their families even though they were the only ones in a position to do so. However, with the introduction of CT, this exploitation reduced. Since CT enabled women to contribute some income to the household, AGYW’s relationships with their spouses improved. AGYW further described how income from CT addressed their difficult economic situations, empowering them economically.

Now if you have your own business the man must respect you, that my wife has got money. …Now that I have my money, even if the man doesn’t provide, you are able to take care of that. (IDI, 19-years, married)

Within the context of intimate relationships, the majority of AGYW (married and unmarried) talked about previously being unable to make decisions in sexual relationships, for fear of losing a partner and his economic support. They reported that they found it difficult to question their partners or suggest to use condoms, even in situations where they knew they were at risk. However, by participating in the CT, they felt empowered to make decisions such as using condoms, reducing number of partners, abstaining from sex, accessing HIV testing, and reducing their engagement in transactional sex. Reflecting on her confidence to negotiate for condom use, one participant stated:

On the issue of using a condom, it is now upon you to decide as we have now been empowered…you don’t depend on him anymore. Previously, they (men) would refuse to use a condom while you (girl) want to use it…he knows that even if you refuse to have sex, you will eventually go back to him with your problems (needs)…. He then reminds you, ‘I told you to do that thing (sex) but you refused’…Now it becomes like a revenge and it is upon you to agree or refuse…but now you know that you have your own business (income), you don’t depend on him for needs… So, if you tell him to use a condom and he refuses, you also refuse (to have sex with him). (FGD)

At the intrapersonal level, AGYW reported increased confidence, self-esteem, and reduced stress as a result of being able to provide for their economic needs. They mentioned that they felt confident about their voice in their families, were self-reliant, and were able to afford and own things they could not previously possess such as mobile phones:

After the cash transfers our abilities have widened…we have received phones so we have been given the ability of becoming strong and confident., CT have also enabled us to believe in ourselves. (FGD)

Further, describing how CT had given her a ‘voice’ and confidence to engage her partner in discussions, a married woman reported:

Most married women, such as myself, didn’t have a voice in our families. I didn’t even know how to stand up to a man or how to talk to him because I was afraid of him. But after this project, I am no longer afraid of him. If there is an issue we discuss. (IDI, 22-years, married)

### Pathways Through Which Participation in Cash Transfers Empowered AGYW

**Fig. 1:**
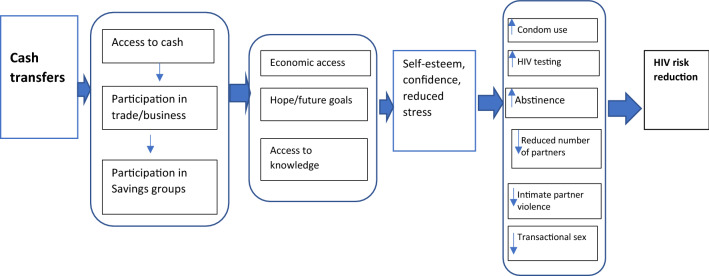
Pathways through a cash transfer intervention empowered AGYW to reduce HIV risk

Based on the themes that emerged on the impact of CT on the lives of AGYW, we developed a framework (Fig. 1) that proposes the potential pathways through which CT empowered AGYW, and how that empowerment impacted their sexual behaviour and could ultimately lead to reduced HIV risk. These pathways are economic empowerment, enhanced hopes and future aspirations, and knowledge.

### Pathway of Empowerment: Economic (Access to Cash for Everyday Needs)

Regular access to cash and freedom to spend was something that AGYW valued. The majority of AGYW reported that, prior to the introduction of the CT intervention, they did not have access to regular cash and struggled to meet most of their needs. CT enabled them to purchase items they desired without depending on sexual partners.

For example, you find that she needs a smart phone but she does not have the money to afford it…Previously, she would go to her partner and talk to him using nice words, so that she can get that money…but since we received money from the CT project and started small businesses and have been getting profit from it…that money multiplied…so now she can do anything that she desires to. (FGD)

Since they now had access to cash, AGYW discussed how they were now able to plan their expenditures and use money wisely on investments, rather than items like clothing. When asked how she made her decision to spend different amounts of CT on different things, an 18-year-old, single woman reported:

It was like I was balancing…I saw it was better to use a large amount of money on something valuable like a business, rather than playing with it…going to buy clothing or maybe you use it on luxury, like eating and drinking. (IDI, 18-years, single)

AGYW reported that they increasingly participated in economic production as a result of the entrepreneurial skills (e.g. soap making) they had learned from the *Sauti* project.

This CT program has helped us. Apart from helping me and my husband it helps me at home. Life at home isn't bad now…Before, I was just a housewife, but the money and the entrepreneurial lessons from this programme like making soaps has helped us. (IDI, 24-years, single)

Another key feature of empowerment observed in the narratives of AGYW was the mention of a shift from dependence on parents and/or partners to self-dependence. The majority of girls did not have their own income and thus were unable to meet their own personal basic needs. This led them to engage in sex, sometimes with multiple partners, some of whom they did not feel emotionally attracted to. Reflecting on their sexual lives, many AGYW talked about how prior to the introduction of CT they had multiple partners but have now reduced this to one partner because they can now meet their own economic needs.

In the beginning I was depending on sex, but now I depend on myself…At first girls had about three to four partners. It is not that they loved them all…but it is just that they meet your needs …they (partners) give you little funds…if you need this or that they would give you…but when we received this money (CT funds), even these issues of relationships reduced…because you realize that even the money he gives you, you can also get it on your own. So, you decide to reduce the partners to just one. (FGD)

AGYW talked about self-awareness and having discovered their potential and ability to look good without depending on men.

Now we are totally self-aware… we can work for ourselves and can look good on our own without depending on someone else…We are doing different activities…Even the community appreciates that this organization has greatly educated our daughters. (FGD)

While expressing worry that the CT were soon coming to an end, married women described the independence that the CT program had brought to their lives by enabling them to take care of some of the family needs and not necessarily ask husbands for everything:

…Some of us never imagined we could become financially independent because we depended on men… But right now, my sister, I can’t hide it from you, right now I am with my business, I don’t depend on a man it really helps us. (IDI, 21-years, married)

AGYW reported that depending on partners for their needs was stressful, and that financial empowerment through CT improved the quality of their relationships with their partners. AGYW attributed their experiences of increased respect, reduced spousal violence, reduced sexual harassment from sexual partners, and increased control in sexual relationships to their participation in the CT programme. Specifically, many married AGYW reported being respected and experiencing reduced spousal conflict after they started contributing to the economic functioning of the household.

Since I started receiving money my relationship with my partner has improved. Before the introduction of the CT, we were fighting because of my needs. We could fight when he has no money. I had a hard time before I started receiving that money. (IDI 19-years, married)

### Pathway of Empowerment: Hope and Aspiration

AGYW described feeling confident and believing in themselves. They reported being able to do what they could not usually do and had never imagined possible for themselves. They were able to spot and utilize opportunities available to them, and engaged in activities that were usually outside their imaginary domains.

The reality is that it [CT programme] has given us the bravery to search/find because we have our capital and are searching/looking; we are doing businesses and are satisfying our small needs. They have given us the courage to run different businesses. (FGD)

One young woman talked about her experience and enhanced hope:

The good things about this project is that it has made things easier for me, so right now I am an entrepreneur…this is something good because before I didn't imagine myself having a business. (IDI, 16-years, single)

Prior to the introduction of the CT and accompanying financial management training, most AGYW did not know the options available to them in terms of lending and borrowing money from existing structures in their communities. With the introduction of CT, many opted to join saving and lending groups that were managed by *Sauti* Project, and were now able to save, lend, and borrow money at some interest.

Honestly, I am grateful for this program, firstly I didn’t know how to save and borrow money, but now, I monitor myself, honestly, I was also dependent like my fellow here. After entering the CT, I got money and started rearing livestock. (FGD)

AGYW reported that they had realized the value of belonging to savings groups because they had a place to go back to in emergency situations.

Eeh, many young women did not know the meaning of savings and economic issues. But they are now part of groups. They can both save and borrow money to start businesses or for other family needs (FGD)

Many AGYW described the way their hopes and aspirations for the future had been enhanced. They talked about the CT as a stepping stone to a much bigger business investment, a “good life” and a “good future.” Many aspired for financial independence, a big business, and having their own family and home. One 17-year-old adolescent girl reported:

This income generation is helping me. I want to put more effort into it so that I can be successful, so that I can be able to have my own family, build my family and my home and have my good life…those are my dreams. (IDI17-years, single)

As indicated in the above excerpt, AGYW felt confident about reaching their goals in life. They expressed changed aspirations, from previously aspiring for very little things such as clothing to now aspiring for things they had never thought about such as owning land. As a result of the success they had in their business investments, AGYW aspired for a “good future”. Many talked about moving from one business to another with an intention of doing something that was profitable.

The decision to save was motivated by the desire to invest in the future. Savings goals included expanding business investments, increasing livestock holdings, purchasing a plot of land, and eventually building a house. One unmarried young woman described her plan for building savings towards personal goals:

When I joined the CT, I started a business and I was able to support myself with my needs. I can now buy my own body lotion, dresses and shoes because I generate profit from the business and my capital is always there… I have now reached my goals and only left with buying a piece of land. My plans are that from doing this business, I will buy a piece of land build a house of my choice in future. Doing my business, I believe I will buy land and then build a house of my choice… I have started paying for the land by installment. (IDI, 18-years, single)

### Pathways of Empowerment: Access to Knowledge

AGYW reported that the CT intervention had empowered them through provision of education on safe sexual behaviour, recognizing violence in sexual relationships, and understanding the need for HIV testing.

For example, through that behaviour change education that we received from the programme…we have been empowered to a large extent. For example, a person like me did not understand the meaning of gender violence…when I heard of gender violence, I assumed that this was only serious things such as a person being slaughtered. But I have now realized that even if a woman is beaten by her partner, that is also gender violence...because he has been cruel to her…he has abused her, he has spoiled her body. (FGD)

Describing the knowledge acquired on the importance of HIV testing, AGYW reported:

We’ve been empowered concerning our big disease (HIV). People are now getting tested; they are really volunteering to get tested than before. In the past it wasn’t that easy for one to tell the other to get tested but now people are getting tested in large numbers. (FGD)

## Discussion

We set out to explore AGYW’s understanding of empowerment, manifestations of empowerment, and pathways through which CT works to empower AGYW economically as well as in sexual decision-making. The three key defining elements of empowerment were access to cash, ability to be independent, and having future aspirations. Our results show that AGYW’s knowledge and understanding of empowerment very much revolved around financial independence, economic achievement, and decision-making in different spheres of their lives. The ways participants described empowerment resonated with the conventional definition of empowerment, which lays emphasis on independence and autonomy [21, 22].

Economic empowerment was linked to AGYW’s ability to participate in economic production, savings groups, and taking care of their needs. Evidence points to AGYW being at high risk of sexual and reproductive health (SRH) problems as a result of their economic vulnerability [4, 23–30]. The practical worth and value of money as an empowerment element in enabling women to make choices and actions has been illustrated in other research in sub-Saharan Africa [31]. Programmes that tackle AGYW’s economic vulnerability clearly have potential for reducing these risks. Structural interventions, including those similar to the CT that was implemented in our study setting, have shown a positive impact on the economic vulnerability of AGYW [17, 32]. The fact that many AGYW in our study were able to meet their basic needs and reported less risky sexual behaviors after CT is encouraging and could point to the need to scale up similar programmes in other contexts in sub-Saharan Africa.

Empowerment manifested at three interlinked levels: community, interpersonal (family and intimate relationships), and intrapersonal levels. AGYW’s access to resources and participation in community savings groups enhanced their solidarity and elevated their social standing at the community level. Inasmuch as a woman’s economic empowerment depended on her position in the family, it determined her control over resources, what she could do outside the family, and ultimately the decision-making power she wielded in her sexual relationships. Increasing women’s social standing in the community could potentially lead to less exploitation and more investment in women. Future studies should examine community-level impacts of CT programmes.

At the interpersonal level, CT influenced AGYW’s intimate relationships with many reporting reduced spousal conflict, partner respect, and increased agency and decision-making power. Economic stressors have been found to increase risk of gender-based violence at the family level [33]. The fact that many AGYW reported being able to provide for the needs of their families and therefore reduced family conflict has implications for the prevention of gender-violence but also for the gender norms around male provision. Despite previous concerns that CT could shift power dynamics in relationships and thus threaten men’s power/control leading to more violence [34], we found no evidence from our findings that CT led to an increase in gender-based violence. AGYW increasingly provide financially and participate in decision-making in their families, a function that was traditionally a male domain [31]. AGYW’s ability to make decisions in their sexual relationships, especially those decisions related to the use of condoms, HIV testing, reduction of number sexual partners and even refusing sex when the partner did not want to listen to them, were strong markers of their empowerment. The fact that some AGYW were able to provide for the economic needs of their families elevated their decision-making positions within their families, especially those who were married. AGYW mentioned that their partners were listening to them more than when they depended solely on them for their needs. This is an attestation to how the CT empowered AGYW in their families. However, as more and more women are able to contribute to the overall economic functioning of their households, it will be interesting to explore the impact of such provision on family and spousal dynamics.

Inasmuch as some AGYW received material support from their families, this was not always sufficient leading to AGYW’s dependence on sexual partners to supplement this. Some studies have noted that it is not easy for low-income households to meet all the needs of AGYW, as some needs may be regarded as luxuries in comparison to other competing household needs [35, 36]. Poverty exacerbates AGYW’s SRH risk, including risk for HIV, as it often heightens gendered power imbalances by pressuring young women to engage in transactional sex [32, 37, 38]. Individual CT to AGYW in our study reduced these behaviours, as the majority reported feeling more financially secure and able to meet their needs through their own cash. The feeling of economic empowerment depicted by AGYW in our study is important for their self-esteem and decision-making, which are in turn key for safe sex behavior. Moreover, girls who are determined to meet their future goals and aspirations can feel inclined to adopt less risky sexual behaviour by making decisions to reduce their number sexual of partners and reduce engagement in transactional sex, as they can now cater for their basic needs [15]. Our results show a change in AGYW’s views on number of partners and desirable qualities for partners. Other studies have observed the main quality AGYW looked for in a sexual partner as being able to provide for them financially, often achieved through transactional sex [35]. In our study, AGYW’s dependence on partners seemed to be changing as many reported new financial independence. Many AGYW increasingly looked beyond a partner’s possession of material resources to qualities such as having a future-oriented mindset, being respectful, and being willing to test for HIV and use condoms. The fact that many AGYW reported changes from risky sexual behaviour to less risky behaviour is encouraging and has important implications for AGYW’s quality of relationships and HIV risk reduction programmes.

At the intrapersonal level, CT gave AGYW a sense of future orientation that encouraged them to aspire for bigger developmental goals such as owning land, a home, enlarging their business; these are things many had never imagined possible prior to the introduction of the CT. As noted in this study, aspirations and feelings of independence are non-tangible benefits that are important to adolescent social growth. Evidence shows that attributes such as independence and social growth are important components of adolescent development, as they are associated with transitions from childhood to adulthood and could lead to improved future aspirations [18].

Whilst all AGYW talked about a feeling of independence after receiving CT, the extent to which this independence influenced economic decision-making varied by marital status. Married AGYW talked about being currently able to make expenditure decisions in the household. Women’s control over resources has been found to affect their control over decision-making in households [11]. While our results on control and decision-making are in line with other authors’ observations [11], this does not align with some other studies, which found that transferring cash to women does not necessarily imply increase in women’s control over these resources [39]. Control over sexual decisions as a result of having access to financial resources might therefore differ between married and unmarried AGYW. Given the growing popularity of CT programmes and widespread interest in women’s empowerment, it is of inherent value to assess whether resources transferred to AGYW through CT programmes are in fact effective in improving their positions within the households and for the unmarried, their decisions in sexual relationships. There is a need for additional longitudinal follow-up with AGYW who received the CT to understand their level of decision-making and control over the resources they had accrued from the CT, and whether that is leading to sustained economic, social, and SRH benefits as reported by AGYW.

A key strength of this study is the longitudinal nature that allowed for exploration of change over time and the use of multiple qualitative methods to triangulate findings. However, similar to any research, this study has limitations. Since this study recruited participants from an ongoing economic empowerment intervention, the *Sauti* programme, there is potential for social desirability bias. It is possible that participants may have reported more positive influences of the programme than negative. This was, however, minimised by having the research done by an organisation independent of the implementing organisation/team. The research team also spent some time with the study participants building rapport and trust in order to encourage participants to give honest responses.

## Conclusion

CT empowered AGYW by fostering more independence and giving them hope and aspirations for the future. The feeling of independence allowed AGYW to use money the way they wanted, participate in economic production and save in ways that made sense to them. A sense of responsibility developed through engagement in business activities, enhanced participants’ self-esteem and confidence in decision-making. This empowerment led to changes in AGYW’s sexual risk behaviors, giving women more authority to negotiate safe sexual behaviors, reduce their number of partners, and not rely on transactional sex.
